# Greater Microbial Translocation and Vulnerability to Metabolic Disease in Healthy Aged Female Monkeys

**DOI:** 10.1038/s41598-018-29473-9

**Published:** 2018-07-27

**Authors:** Quentin N. Wilson, Magan Wells, Ashley T. Davis, Christina Sherrill, Matthew C. B. Tsilimigras, Roshonda B. Jones, Anthony A. Fodor, Kylie Kavanagh

**Affiliations:** 1 Wake Forest School of Medicine, Department of Pathology, Winston-Salem, USA; 2 University of North Carolina at Charlotte, Department of Bioinformatics and Genomics, Charlotte, USA

## Abstract

Monkeys demonstrate gastrointestinal barrier dysfunction (leaky gut) as evidenced by higher biomarkers of microbial translocation (MT) and inflammation with ageing despite equivalent health status, and lifelong diet and environmental conditions. We evaluated colonic structural, microbiomic and functional changes in old female vervet monkeys (*Chlorocebus aethiops sabeus*) and how age-related leaky gut alters responses to Western diet. We additionally assessed serum bovine immunoglobulin therapy to lower MT burden. MT was increased in old monkeys despite comparable histological appearance of the ascending colon. Microbiome profiles from 16S sequencing did not show large differences by age grouping, but there was evidence for higher mucosal bacterial loads using qPCR. Innate immune responses were increased in old monkeys consistent with higher MT burdens. Western diet challenge led to elevations in glycemic and hepatic biochemistry values only in old monkeys, and immunoglobulin therapy was not effective in reducing MT markers or improving metabolic health. We interpret these findings to suggest that ageing may lead to lower control over colonization at the mucosal surface, and reduced clearance of pathogens resulting in MT and inflammation. Leaky gut in ageing, which is not readily rescued by innate immune support with immunoglobulin, primes the liver for negative consequences of high fat, high sugar diets.

## Introduction

There is loss of gastrointestinal (GI) mucosal barrier function (described as “leaky gut” in lay terms) with age in invertebrates, animals, and people^1–3^. A leaky GI mucosa allows greater translocation of microbial antigens (microbial translocation; MT) into the intestinal wall and portal circulation, inciting inflammation locally and remotely^4^. MT is potentially important in driving metabolic health, as there is evidence linking bacterial endotoxemia to the accumulation of ectopic fat^5–7^, which is an important driver of insulin resistance^8^. Although intestinal leakiness occurs with advancing age across multiple species, it has only recently been additionally documented as an important mediator of lifespan, at least in invertebrates^2^.

Trends in microbiomes of aged animal models or people have not been consistently observed^9,10^. Clinical studies have repeatedly demonstrated geographic, dietary, and medication effects on the microbiome, making generalizations about age difficult. Most studies evaluate the fecal microbiome, which represents a “catch-all” approach for estimation of colonic contents and may not represent location-specific microbiomes. The microbiome in close association with the mucosal surface may differ, and may be a small sub-population of the general microbiome that cannot be captured with gross fecal analysis^11,12^. Differences may be attributed to effects of the intestinal epithelial cells, which are constantly interacting with the microbiome. The mucosa contributes to host defense through several mechanisms, with immune competence being closely related to mucosal barrier function^12,13^. It is well appreciated in human and non-human primates that the elderly experience immunosenescence^14–16^, and currently there are no approved therapeutic strategies that target immunosenescence, specifically MT, or have indications for ageing, although prebiotics and probiotics are commonly used over the counter strategies.

Age-related increases in leakiness are difficult to assess in human clinical trial settings as age, diet, and environmental factors in people typically change concomitantly. In the current series of studies we aimed to survey the structural and functional changes of the gut with ageing, using a relevant non-human primate model that is not confounded by medications or dietary influences^17,18^. We hypothesized that the GI barrier defects that we have observed in older monkeys^10^ may be improved with oral dosing with serum bovine immunoglobulins (SBI) which is a clinically utilized non-absorbable dietary protein product that consists of multiple immunoglobulin classes from a large pool of bovine donors. It thus captures immunoglobulin types generated from a wide range of pathogens. These immunoglobulins enable some direct binding of pathogens and their toxins, whereas the Fc region of the protein interacts with immune cells that support intestinal homeostasis to reduce inflammatory stimuli and MT^19^. It is believed that its predominant effect is enhancement of intestinal barrier integrity rather than endotoxin binding to reduce MT^20^.

We report herein that age-related leaky gut occurs with age and that Western diet challenge does not further augment leakiness, but does lead to exaggerated declines in health. Our data suggests that adequate colonic physical barrier elements and innate immune responses to MT are present in old monkeys, and thus it may not be surprising that additional oral immunoglobulin therapy was not effective in reversing leaky gut or improving health. The microbiome profile was similar across ages, and not considered to be a major driver for loss of mucosal barrier function. Age-related adaptive immunosenescence should be investigated as a mechanism and target for improving gut function in the older population.

## Results

**Figure 1:**
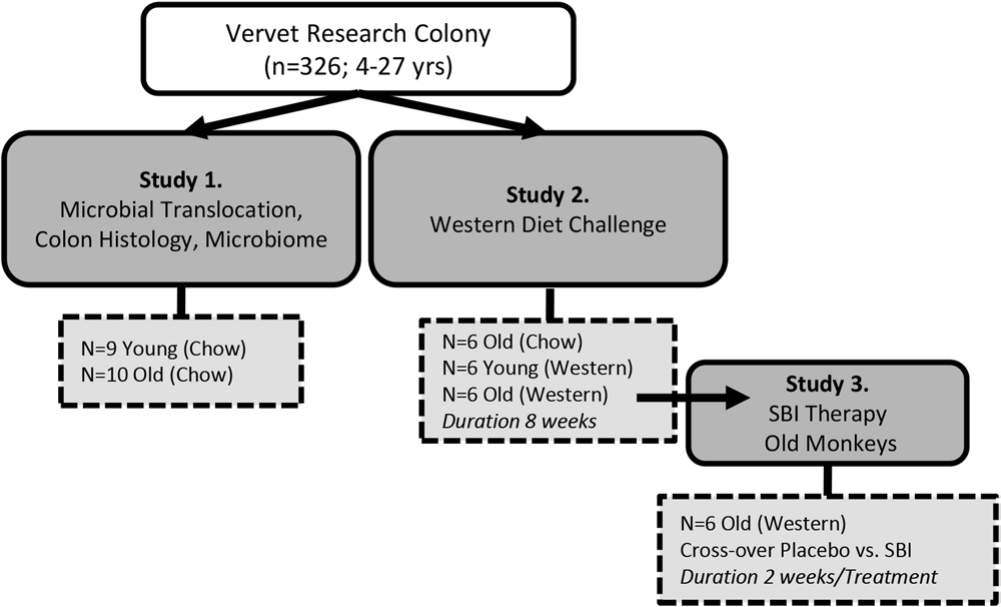
Overview of monkey cohorts described within this report.

An overview of studies and the monkey groups are depicted in Fig. 1.

### Study 1. Microbial translocation, colon histology, and microbiome of healthy young (n = 9) and old monkeys (n = 10)

**Figure 2:**
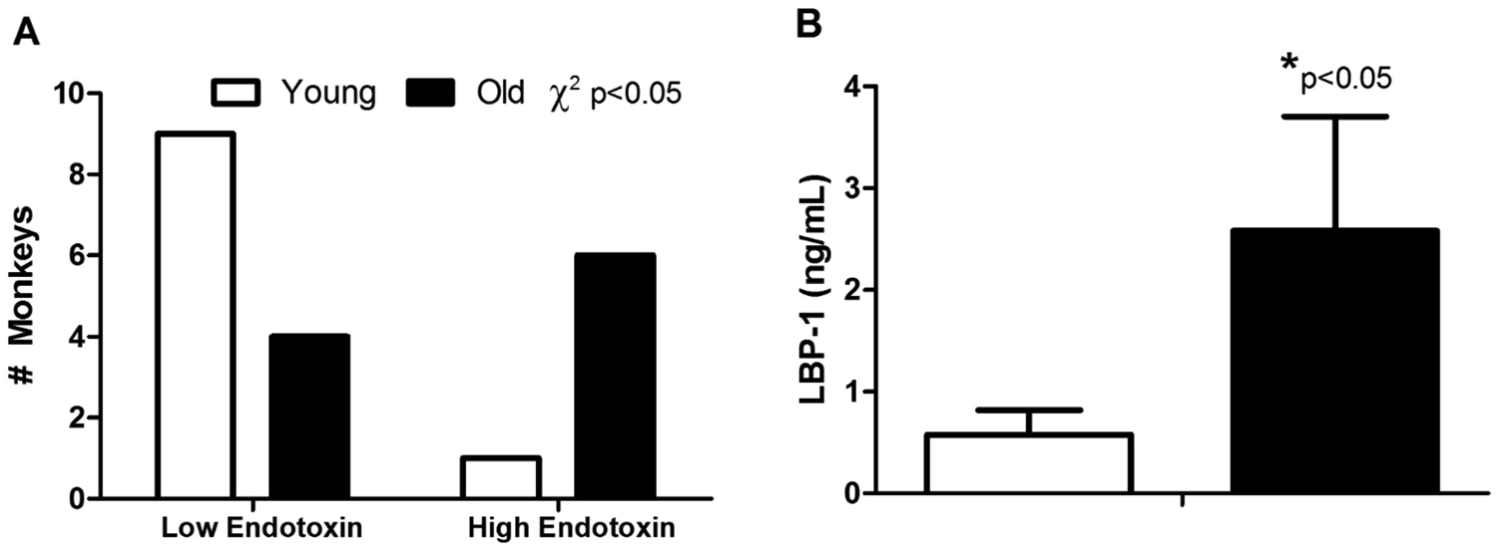
Microbial translocation was higher in old monkeys despite lifelong environmental and dietary equivalence and good health. Endotoxin levels detected in portal vein plasma were more frequently elevated in old monkeys (Top Panel A). Lipopolysaccharide binding protein 1 (LBP-1) was also higher (Bottom Panel B). Results are from monkeys described in Study 1 (n = 9 young and n = 10 old monkeys).

**Figure 3:**
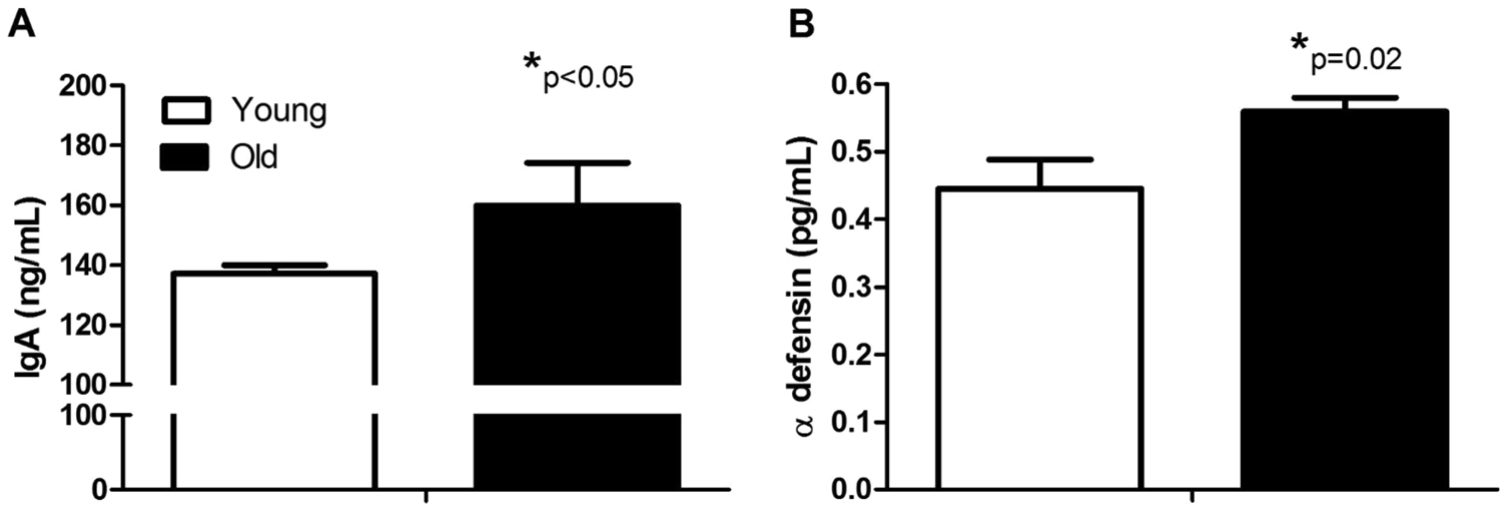
Non-specific immune responses to microbial translocation are higher in old monkeys. Plasma levels of secretory immunoglobulin A (IgA; Top Panel A) and antimicrobial peptide, alpha defensin (α-defensin; Bottom Panel B) are significantly different in young and old monkeys. Results are from monkeys described in Study 1 (n = 9 young and n = 10 old monkeys).

Subject’s health characteristics are shown in Supplementary Table S1. Old monkeys were in good health but had weight redistribution leading to a higher waist circumferences, however values are all within the normal, non-obese range for this species and this specific population^21^. Chow consumption was assessed in a separate cohort of age-matched young (n = 22) and old monkeys (n = 11). The monkeys were sourced from the same vervet colony and showed no significant differences in daily intake (104 vs. 104 g/day, p = 0.33). MT rates in old monkeys were significantly elevated as portal vein endotoxin levels, a direct measure of translocated Gram-negative bacterial product, had a distribution that indicated higher amounts in the blood draining the gut and travelling to the liver (Fig. 2A). Accordingly LBP-1, which is produced in the liver as a result of endotoxin exposure, was nearly five-fold higher in old monkeys (Fig. 2B; p < 0.05 for both endotoxin and LBP-1). sCD14 levels were not different between young and old monkeys in this cohort (845 vs 734 µg/mL; p = 0.73). The histostructure of the colon did not differ with age (Table 1). Innate immune responses, measured as circulating secretory IgA and antimicrobial peptide, α-defensin 5, were both greater in old monkeys by 16% and 25% respectively (p < 0.05 for both), consistent with responses to a leakier gut (Fig. 3A,B).

**Table 1:** Histological parameters measured from young and old monkeys evaluated in Study 1.

	Young (n = 9)	Old (n = 10)	p-value
Crypt depth (µm)	320 (22.7)	321 (11.35)	0.49
Goblet cells (#/100 µm)	12.31 (0.46)	12.51 (0.60)	0.49
Mucus staining (% area)	71.40 (3.71)	71.32 (2.53)	0.99
Muscularis layer thickness (µm)	801 (93)	799 (88)	0.39
Occludin staining (% area)	3.77 (0.50)	4.10 (0.59)	0.67
Claudin staining (score)	1.95 (0.28)	3.00 (0.53)	0.08

Values are mean, with the SE shown in parentheses.

**Figure 4:**
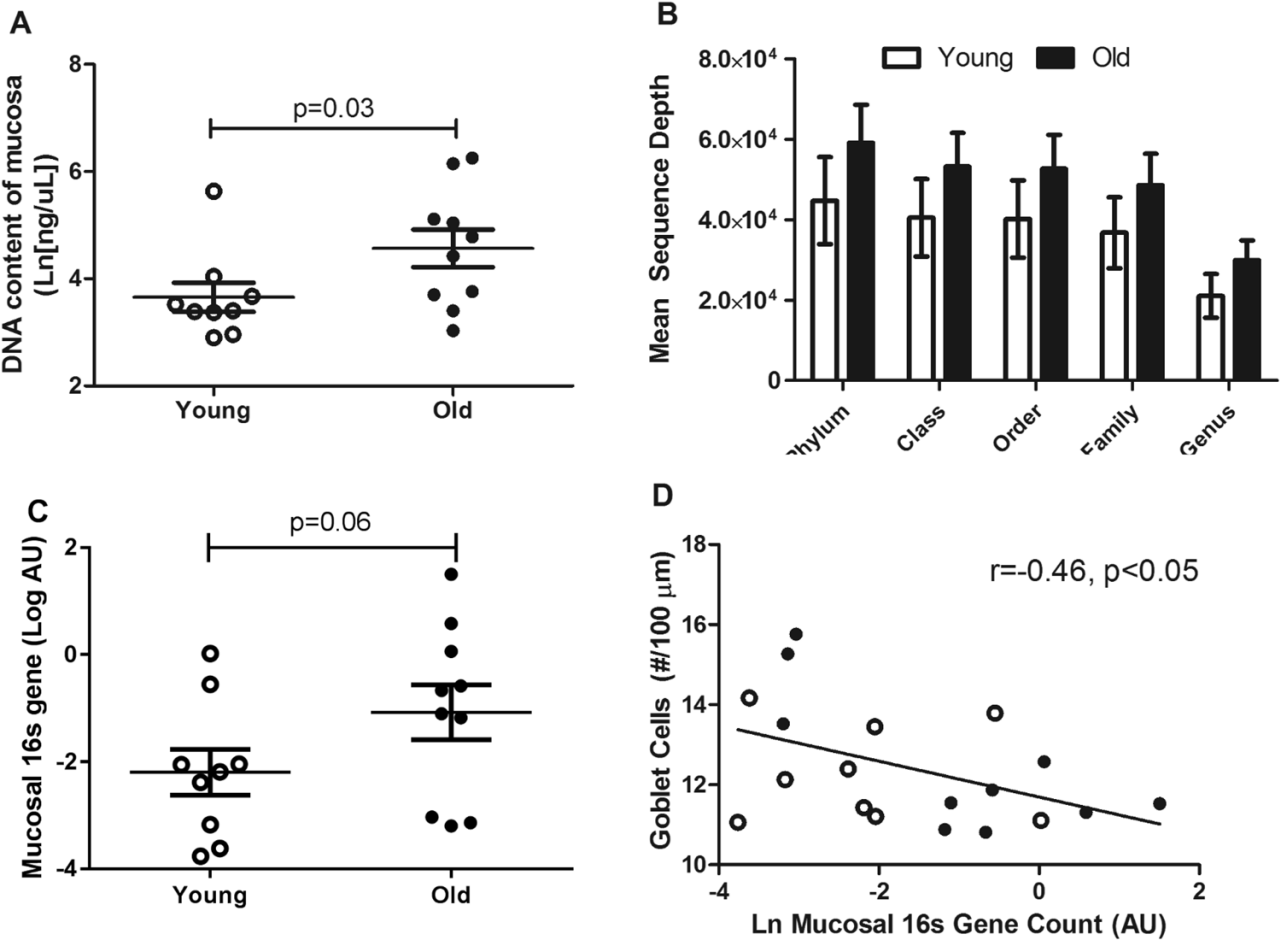
Old monkeys had higher bacterial loads detected from samples retrieved from the mucosa of the ascending colon. The DNA content of the mucosal samples was greater in old monkeys (**A**). The DNA was likely related to the presence of more bacteria as mean sequence depth by Illumina DNA sequencing identified to bacterial taxa (**B**) were approximately 1.35 fold higher in old monkeys, and PCR-measured total bacterial 16S gene counts normalized to tissue weight (**C**) were more than doubled on average in old monkeys. The colon mucosa is protected by a mucus layer which physically separates the microbiome. Goblet cells, which produce mucus, relate to bacterial gene counts on the mucosal surface such that more goblet cells were associated with fewer bacterial genes (**D**; young monkeys are open circles [○] and old monkeys are closed circles [●]). The microbiome profile did not differentiate by age in monkeys consuming the same diet and exposed to the same lifelong conditions (Panel E; LI = large intestine). Results are from monkeys described in Study 1 (n = 9 young and n = 10 old monkeys).

Mucosal samples from old monkeys had higher DNA content (Fig. 4A). Microbial abundance on the mucosal surface trended higher in old monkeys (Fig. 4B,C). Average bacterial DNA sequence depth after amplification was higher in older monkeys (34% greater), consistent with the greater bacterial loads in mucosal scrapings seen by qPCR. While none of these measures are perfect indicators of total bacterial number, together they support a hypothesis of higher loads on the aged colon mucosa. Goblet cells produce protective mucus which physically separates bacteria from the cells of the gut barrier and an inverse relationship between the bacterial abundance and goblet cells was also observed (Fig. 4D). There were no differences by age grouping in the amount of mucus detected within goblet cells of the colon (Table 1). The microbiome profiles across the 3 sampling sites were not significantly different between the young and old monkeys, which was expected based on the consistent diet and our prior evaluations from the same colony (Fig. 4D; Supplementary Tables S2–S4)^10^. Diversity of microbiome profiles differed by sampling location, with the mucosa being significantly less diverse than the feces or colon lumen content at the phylum level (p < 0.001 and p = 0.004 respectively after post-hoc Tukey’s honest significant difference test; Supplementary Fig. S1). Similarly, there were no statistically significant associations between the relative abundances of individual taxa and the numeric age or the age group of the monkeys at a false discover rate of 0.05.

### Study 2. Response to Western diet challenge in young and old monkeys

**Figure 5:**
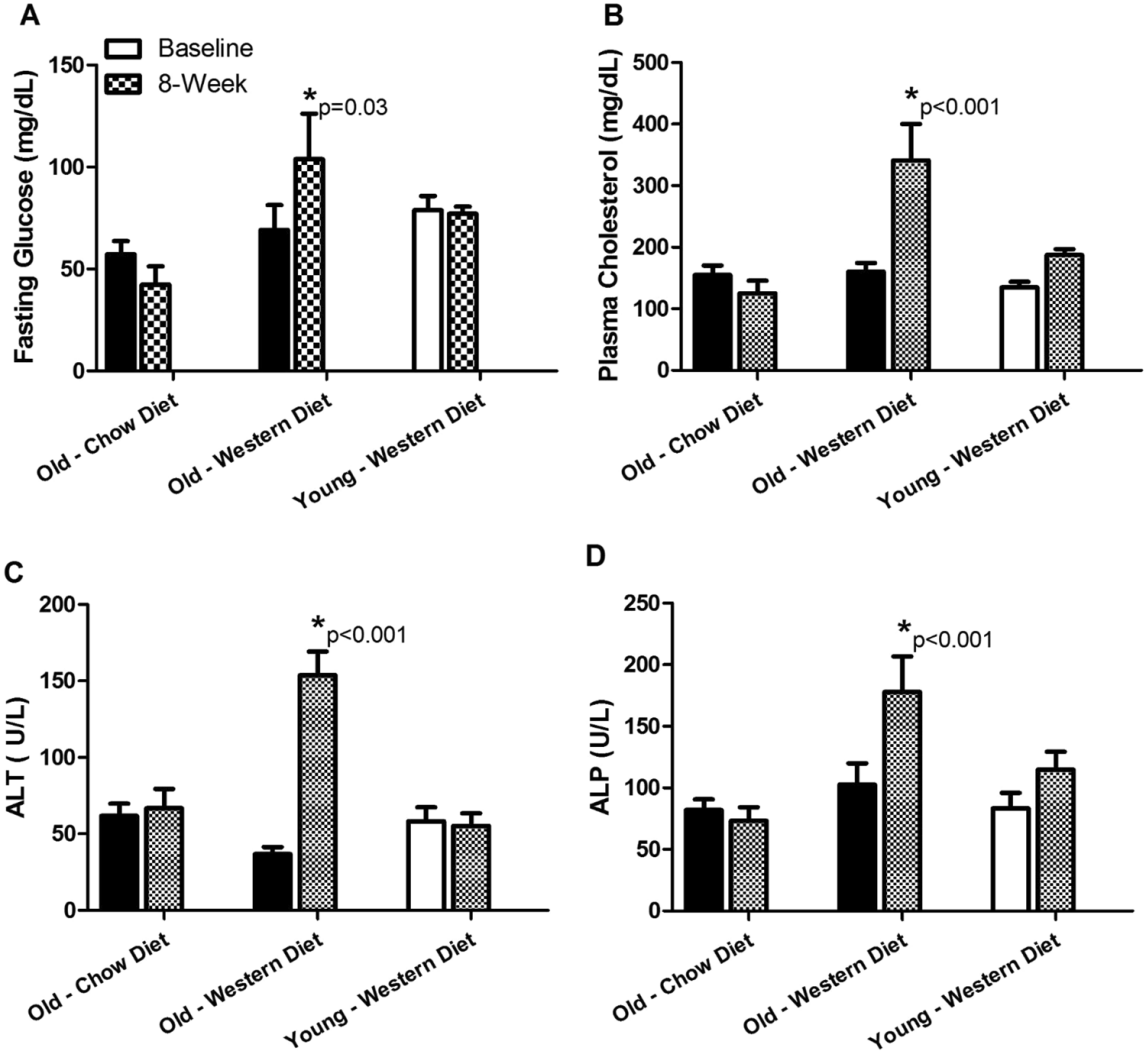
Old monkeys had significant deleterious health effects when challenged with 8 weeks of a Western diet. We observed that old monkeys significantly increase plasma glucose (Panel A), cholesterol (Panel B), and liver enzymes alanine aminotransferase (ALT; Panel C) and alkaline phosphatase (ALP; Panel D) whereas young monkeys were able to maintain homeostasis under the same dietary challenge conditions. Old monkeys maintained on a chow diet showed no changes over time. Old monkeys are shown with black bars, young monkeys are shown in white, and the post-diet challenge value indicated for each group by checkerboard. Results are from monkeys described in Study 2 (n = 6/group).

Baseline data from the 18 animals included in this study is shown in Supplementary Table S6. Six young and 6 old monkeys transitioned to a Western diet (Supplementary Tables S5 and S6) for 8 weeks, while 6 additional old monkeys stayed on the chow diet after baseline assessments to control for changes over time. At the end of the diet challenge old monkeys exhibited significantly elevated liver enzymes, plasma cholesterol, and glucose values, whereas young monkeys consuming the Western diet and old monkeys remaining on chow were unaffected (Fig. 5). Healthy old monkeys went from having normal to impaired fasting glucose values (>100 mg/dL fasting glucose). Five of the 6 old monkeys were available to re-evaluate ALT values 8 weeks after return to the healthy chow diet, and notably ALT remained significantly elevated in 3 of the 5 animals (Supplementary Fig. S2). This indicated that old monkeys are both less resilient to the nutritional stress of a diet challenge, and individuals are susceptible to persistent hepatitis. Cholesterol increased in both young and old monkeys consuming the Western diet, but more than doubled in value from baseline in the old monkeys (p < 0.001) as compared to more nominal changes in young monkeys (p > 0.05) (Fig. 5B). MT markers were measured in Western diet-fed monkeys and they did not further increase in old monkeys, while young monkeys’ values did not change significantly in response to the 8-week diet challenge (Supplementary Table S7). We did not measure the microbiome, as prior diet studies have shown this intervention is not long enough to enable the detection of stable changes in fecal profiles with this number of subjects^4,22^.

### Study 3. Serum Bovine Immunoglobulin (SBI) therapy in old Western diet-fed monkeys

Old monkeys (n = 6) were given placebo or 6 g/day of SBI daily added to 80 g of their daily food allotment for 2 weeks in a cross-over study, with 4 weeks of washout between treatment periods. There was with 100% compliance in all monkeys orally consuming the SBI doses, verified by direct observation of dose consumption. No changes in food intake or fecal characteristics were observed. SBI was not effective (Table 2) in changing health related parameters after 2 weeks of dosing. MT markers remained high as did hepatic and metabolic markers.

**Table 2:** Selected outcome variables measured in old monkeys after placebo or 2 weeks of treatment with serum bovine immunoglobulins (SBI).

	N	BW	Waist	Glucose	TPC	ALT	ALP	LBP-1	sCD14
		kg	cm	mg/dL	(mg/dL)	(U/L)	(U/L)	ng/mL	ng/mL
Placebo	5	5.38 (0.25)	35.4 (0.40)	95.6 (25.3)	349 (64.5)	143 (15.5)	145 (28.8)	4.47 (0.88)	2.47 (0.21)
SBI	6	5.43 (0.30)	36.3 (1.01)	118 (23.2)	301 (64.2)	159 (51.5)	178 (31.1)	5.67 (0.96)	2.66 (0.17)
p-value		0.27	0.28	0.23	0.44	0.06	0.66	0.53	0.58

Values are mean, with the SE shown in parentheses.

## Discussion

Our studies facilitate an attempt to isolate age-related factors from environmental influences in a primate GI system. In doing so, we confirm that healthy ageing is not associated with large structural changes in the colon as has been discussed across models and clinical studies^23^, but rather with functional deficits in permeability of the GI barrier. We posit that age-related increases in GI permeability (leaky gut) may be linked to greater numbers of resident bacteria on the mucosal surface. The main functions of a competent intestinal mucosal barrier are the regulation of bacterial numbers and types residing on the surface, and clearance of pathogens detected in submucosal sites and reticuloendothelial tissues on the serosal side of the gut. Bacterial clearance mechanisms could be overwhelmed or compromised by immune deficiencies that occur with normal ageing^16^. MT and health is improved in antibiotic-treated or germ-free aged rodents, which supports overgrowth of bacteria on the mucosa as a mechanism for age-related barrier dysfunction^3,6^. We observed that high MT burden in old monkeys primes them for deterioration of metabolic and liver biomarkers in response to diet challenge and underscores the need for attention to diet quality in the aged, as it relates to frailty and health outcomes^24,25^. We also tested 2-week intervention of SBI, which is known to reduce endotoxemia or MT^26,27^, and found it does not provide benefit in aged monkeys.

In the young healthy host, MT occurs at a low rate; however, the immune defenses remove bacteria effectively through processing by the immune cells in the mucosal lamina propria or delivery of bacteria via the draining lymph to regional lymph nodes^28^. Aged mice have lower ability to present antigens from the GI mucosal surface and facilitate their clearance^29^. Excessive infiltration of bacteria or bacterial products into the mucosa in young GI tissues leads to a deregulated intestinal immune response resulting in the development of gastrointestinal disorders^30^. This deregulation effect may be greater, however, in immunosenescent GI tissues of older animals.

Captive monkeys undergo age-related immunosenescence much like that described in humans. Changes are characterized by shifts in immune profiles that include: a loss of naïve T cells with an accompanying shift towards memory phenotype T cells, loss of T cell repertoire diversity, and greater systemic inflammation^31^. Our results show innate immune responses measured as IgA and α-defensin in circulation were higher. Although these biomarkers are not specific to gut-derived antigens, they do demonstrate upregulation in accordance with endotoxemic burden^32,33^. However, there are few studies that evaluate circulating levels of defensin proteins, so their value as biomarkers is not yet fully understood. Defensins are not solely produced by Paneth cells so circulatory changes may not be specific to gut pathology, however their increase does support a greater pathogen load seen with ageing.

The risk for developing metabolic disease and liver damage increases with age in men, women and non-human primates^34–36^. A consequence of Western diet (high calories, high fat, high simple sugars) consumption is liver damage^4,5,37^, and in the monkey model we have shown that both age and diet independently predict the severity of liver disease^38^. Thus we rationalized that advanced age plus Western dietary challenge would interact on GI barrier function to synergistically drive a worsened health outcome. A leakier gut in aged monkeys pre-existed the exaggerated responses to dietary challenge. This is consistent with the concept of “inflamm-ageing” and the observations that metabolic disease, and specifically subclinical hepatitis, is greater in older people^35,36^. When biomarkers LBP-1 and sCD14 already indicate that high MT rates exist, diet challenge did not further exacerbate this biomarker response which is consistent with recent clinical trials comparing the responses of young and old people to a short-term diet challenge^39^. Oral immunoglobulins have been used extensively in veterinary medicine for decades to aid in intestinal support, prevent endotoxemia and reduce inflammation, and thus we hoped that such therapy would be effective in reducing endotoxemia and inflammation seen in ageing^20^. SBI is clinically indicated in HIV-associated enteropathy which, like ageing, is associated with immune deficiency. However, even in HIV patients, an SBI intervention similar to that conducted here did not consistently improve GI barrier function^40^, with positive trials generally being limited to diarrheal disease and colitis^27^. The results presented herein from the monkey studies indicate that immunoglobulin responses are present in response to a leaky gut, and additional immunoglobulins do not improve barrier function. Consistent with the failure of this immunoglobulin approach is the failure of new pharmacotherapies with endotoxin binding capacity (sevelamer is an example) to reduce MT biomarkers^41^. Thus, we suggest that other strategies to reverse immunosenescence, such as inhibitors of the mammalian target of rapamycin or long-term caloric restriction, which are effective enhancers of human and non-human primate T cell function, as potentially being more effective^31,42^.

We focused our analysis on simplified microbiome outcomes – diversity and mucosal abundance. These variables are increasingly being advocated for as measures that can be compared across studies^43,44^. We found that diversity is less at the mucosal site, which is expected as the mucosa favors survival of microbes known to colonize the outer intestinal mucus layer. The presence of abundant protective mucus and the resultant selection pressure to reduce bacterial diversity are both features that prevent metabolic disease development in animal experiments^12,45^. The observed relationship between goblet cells, which produce mucus, and bacterial gene counts at the mucosa support this concept.

Limitations of the current study include the enrollment of only female subjects and the relatively small number of individuals. We have now demonstrated an increase in MT in two separate cohorts of aged monkeys in this paper and in a previously published third cohort^10^. We are confident that the reproducibility of this age-related effect, seen in the absence of dietary challenge or disease, strengthens the subsequent observations relating to the microbiome, effects of diet and SBI interventions. We cannot generalize that aged males will be similar, but sex-effects in MT rates have not been reported. We also cannot imply causality to changes in mucosal barrier function and microbiome. Our model has the advantage of lifelong comparability of environment (including diet, geography, and social contacts) which is known to shape the majority of microbiome profiles. We see microbiome changes in this stable model only after many months of dietary intervention^22^, consistent with what is known about aged humans transitioning their physical and nutritional diet^24^. We know microbiome manipulations can alter permeability in mice^3^, but our data suggests that microbiome changes with healthy ageing are small and limited to shifts in abundance. Such changes could result from reduced immune surveillance, and thus increased MT follows. Our model suggests that antibiotic approaches, pre/probiotic approaches known to enhance mucus, or immune restoration are worthy of pursuing and translating to human health.

The absence of any detectable shift in microbiome profiles between age groups across sampled sites highlights the effects that diet and environmental consistency have on such profiles and allows us to speculate that mucosal pathogen load – rather than changes in community composition - is a key change in the ageing gut. Our SBI outcomes are clinically relevant as it is a cheap and readily available intervention being utilized in enteropathies that have an immune component. In conclusion, the ageing GI is still a relatively unknown landscape. We provide data in a primate GI system that ageing without dietary, comorbid disease, or treatment effects leads to reduced barrier function of the mucosa. The ageing GI may make old primates less resilient to challenges such as poor diet quality. The translational relevance of our study is to shape future investigations into improving barrier function, and disseminating negative findings about SBI.

## Methods

### Animals and Experimental Design

The study subjects were all sourced from a multigenerational pedigreed colony of vervet monkeys (*Chlorocebus aethiops sabeus;* n = 326, 4–27 years, lifespan ≈ 26 years) which descended from 57 founder monkeys (the Wake Forest Vervet Research Colony)^21^. Most females are housed within their natal social group for the duration of their naturally determined lifespan^21^. Due to the nature of a breeding colony, aged males are not retained, so our study populations were all female. Females were classified as either young or old (>18 years), and evaluated them in a series of studies depicted in Fig. 1. Animals in the colon survey study (Study 1) were fed a commercial laboratory primate chow (Laboratory Diet 5038; LabDiet, St. Louis, MO) with daily supplemental fresh fruits and vegetables. This standard laboratory diet comprised of 13% of calories from fat; 69% of calories from carbohydrates; and 18% of calories from protein (Supplementary Table S5). These animals were housed in large indoor/outdoor enclosures that provide elevated perches and climbing structures within multigenerational social groups and *ad libitum* opportunities to socialize, eat, and exercise. Diet consumption patterns were evaluated in a representative selection of young and old monkeys from the same colony by measurement of diet fed and diet remaining over 24 hours. Measures were available on average over 3 different days (range of 1–10 consumption measures/monkey). Blood and tissue samples were collected from Study 1 at a scheduled euthanasia time point, which differed from studies 2 and 3 as anaesthesia was achieved with sodium pentobarbital and blood was terminally collected from the vena cava. A separate selection of old and young monkeys from the colony were challenged with a Western Diet (Study 2; Supplementary Table S5). The Western diet comprised of 35.5% total calories supplied as fat and 14% as saturated fat. Carbohydrates were 46% of total calories with 22% supplied as simple sugars and total caloric density was. Protein content was comparable to the chow diet at 19% of calories, however the protein source was predominantly from animal sources. Dietary fibre was comparable between diets and approximates recommended human consumption levels at 35 g/day. Assessments were performed pre-diet and 8-weeks post-diet initiation. At the 8 week time point, the old monkeys continued on Western diet to evaluate the effects of SBI supplementation (Study 3). Old monkeys (n = 6) were given placebo or 6 g/day of SBI daily added to 80 g of their daily food allotment for 2 weeks in a cross-over study with 4 weeks washout between treatment periods. This dosing duration has resulted in improved MT parameters in animal studies^20,26^. The SBI product used was EnteraGam® (Entera Health Inc., Cary NC), which is a medical food product available by prescription for the treatment of severe inflammatory bowel disease and HIV-associated enteropathy. The dose was chosen to deliver the paediatric dose (4 g/d), while allowing for waste. The dosed food was consumed fully before additional food was offered to the monkeys. Daily observations were made and all monkeys were compliant in consuming the SBI dose. Blood samples were collected at the end of each treatment period. One animal was euthanized such that 5 monkeys were assessed with placebo and 6 monkeys were assessed with SBI treatment. The euthanasia occurred during the washout period following SBI therapy and was necessitated due to a diagnosis severe pancreatitis. Pancreatitis was confirmed by diagnostic pathology on tissues collected at necropsy.

All animal procedures were performed on a protocol approved by the Wake Forest University Institutional Animal Care and Use Committee according to recommendations in the Guide for Care and Use of Laboratory Animals (Institute for Laboratory Animal Research) and in compliance with the USDA Animal Welfare Act and Animal Welfare Regulations (Animal Welfare Act as Amended; Animal Welfare Regulations).

### MT and Metabolic Health Biomarkers

Animals were fasted overnight and anesthetized with intramuscular ketamine (10 to 15 mg/kg) to allow for sample and data collections in Study 2 and 3. Each animal was weighed and waist circumference measured with a flexible tape measure at the level of the umbilicus. Blood samples were obtained by venipuncture of the femoral vein into ethylenediaminetetraacetic acid (EDTA) and serum separator blood tubes. The EDTA anticoagulated blood was held on ice until it could be processed. After processing the plasma and whole blood, samples were stored at −80 °C until analysis. General metabolic health and biochemistry panels were measured as previously described^21,34^. Portal vein endotoxin levels were measured from samples collected in Study 1 coincident with colon tissue and measured by the Kinetic-QCL™ Kinetic Chromogenic LAL assay (Lonza Group Ltd, Walkersville, MD) and results characterized as high (>0.5 EU/mL) or low (<0.5EU/mL) endotoxin levels. In studies 1, 2, and 3, the biomarkers of MT used were plasma concentrations of lipopolysaccharide binding protein 1 (LBP-1) and soluble CD14 measured by ELISA as previously described^10^. Innate immune response to MT was estimated by ELISA detection of circulatory levels of secretory IgA and the antimicrobial peptide, α-defensin 5, which is primarily sourced from gut Paneth cells (eBioscience, Vienna Austria and MyBioSource Inc., San Diego CA). We included liver enzymes alanine aminotransferase and alkaline phosphatase (ALT, ALP) in Studies 2 and 3 as portal delivery of endotoxin is known to initiate hepatic damage in monkeys within this timeframe^38^.

### Histology

Full thickness sections collected from the midpoint between the cecum and of the transverse colon (ascending colon) were collected after euthanasia from each monkey in Study 1. Colon tissue was formalin fixed and paraffin-embedded prior to sectioning and staining with hematoxylin and eosin. Mucus content was estimated by co-staining with Alcian Blue and periodic acid Schiff staining for total proteoglycan content. After initial observation for quality, a blinded observer measured the crypt depth and number of goblet cells along 10 intact villi. Goblet cells are expressed as number per 100 microns. Mucus content was estimated by outlining 10 crypts of intact villi as a region of interest and analysing the percent area stained blue/purple using imaging software (Supplementary Fig. S3; Visiopharm, Broomfield CO). The thickness of the muscularis layer was measured in transection at its widest point. Immunohistochemical staining of the tight junction proteins occludin and claudin-1 was evaluated by color pixilation quantitation by imaging software and expressed as the average area for occludin (Image Pro Plus, Version 5.1; Media Cybernetics), and by scoring staining intensity as 1–5 for claudin-1. Antibodies for occludin and claudin-1 were sourced from Abcam (Cambridge, MA #ab15098 at 1:500) and Santa Cruz Biotechnology (Dallas, TX #133255 at 1:200) respectively, and immunostaining protocols have been previously described^4^. Example images are shown in Supplementary Fig. S3.

### Microbiome

Samples from the 19 monkeys included in Study 1 were collected for microbiome characterization. Feces, colonic lumen content, and a mucosal scraping from the same region of ascending colon were collected from each monkey was used as material for DNA extraction. DNA was quantitated and sequenced by a commercial laboratory (HudsonAlpha Institute for Biotechnology, Huntsville AL). Briefly, DNA was extracted from tissue samples (Qiagen), the quality of DNA determined by gel electrophoresis, and then the amplified DNA was sequenced (Illumina Miseq PE250). Sequences are available at the SRA via accession SRP139357. Mucosally-sourced DNA had total bacterial gene content estimated in duplicate by PCR and was normalized to the tissue weight used as the starting material^46^. Old monkeys had variable 16 s abundance in the mucosa however all values, including outliers, were retained in the dataset as review of laboratory processes and data support these values are being real. Taxonomic assignments from 16 S rRNA sequencing were performed using the RDP classifier (version 2.6) with default parameters with a confidence score of 80%^47^. An additional taxonomic classification using QIIME (version 1.8) for closed-reference operational taxonomic unit (OTU) picking with default parameters against the Green Genes database version 13.5 at a 97% identity to test for consistency between classification methods was performed^48^. The resulting count tables were log normalized as previously described^49^. Ordination by principal coordinates analysis was performed using the Bray-Curtis dissimilarity via the capscale function of the vegan R package. The related R scripts are publicly available through GitHub at https://github.com/mcbtBINF/IntestinalAging/.

Statistical models in R were made for each of the response variables of log normalized bacterial abundance, sample Shannon diversity (representing within-sample alpha-diversity) and ordination axes (representing between-sample beta-diversity). The response variables were investigated on each of the three sample types (feces, lumen, and mucosa) as well as pooling the tissue types together in a mixed linear model. The mixed linear model nested the sample types within the associated animal using the lme function from the R package nlme, in addition to the explanatory variables of “age-group” and sample sequencing depth. Additional models consisted of the nonparametric Wilcoxon test with animals split by “old” and “young” status on each sample type, and a simple linear model with explanatory variables for “age-group” and sample sequencing depth for each sample type. The Benjamini-Hochberg method for multiple hypothesis correction was used with the false discovery rate set to 0.05. A detailed set of microbiome tables and graphs from these analyses is available in the Supplementary Data files.

### Data analysis

Data are presented as means ± SEM for each group. Group sizes for each measure are indicated in figures and tables. Data were analyzed for normality and logarithmically transformed where necessary before AN(C)OVA (with adjustment for baseline value if available as in Study 2) was performed to assess for group differences. Differences in proportions in Study 1 endotoxin levels were analyzed by Fisher’s exact test. Study 3 was a cross-over design and repeated measures ANOVA was used with time, treatment sequence, and baseline values used in the statistical model. Post hoc determinations of group differences were done using Tukey’s honestly significant difference tests. Pairwise associations between variables were evaluated by using Pearson’s correlation coefficient if normally distributed. Statistical analysis was performed with Statistica v10 software (StatSoft Inc., Tulsa OK). Significance was set at α < 0.05 for group differences and α < 0.10 for trends.

## Electronic supplementary material

Supplementary Data
