# Supplementary material for: Greater Microbial Translocation and Vulnerability to Metabolic Disease in Healthy Aged Female Monkeys
Source: Sci Rep. 2018 Jul 27;8:11373. doi: 10.1038/s41598-018-29473-9 (PMC6063974; doi:10.1038/s41598-018-29473-9)

Supplementary Data File

Greater Microbial Translocation and Vulnerability to Metabolic Disease in Healthy Aged Female Monkeys

Quentin N. Wilson[1], Magan Wells[1], Ashley T. Davis[1], Christina Sherrill[1], Matthew C. B. Tsilimigras[2], Roshonda B. Jones [2], Anthony A. Fodor[2], Kylie Kavanagh[1*]

Supplementary Table S1.

Basic demographics of young and old monkeys evaluated in Study 1.

	Young (n=9)	Old (n=10)	p-value
Age (yrs)	10.7 (0.63)	22.1 (0.38)	<0.001
BW (kg)	4.87 (0.41)	4.94 (0.26)	0.45
Waist (cm)	34.3 (2.32)	38.6 (0.83)	0.04
Fasting glucose (mg/dL)*	136 (31)	119 (17)	0.30
Fasting insulin (μIU/mL)	32.5 (12.6)	16.1 (2.87)	0.09
HOMA score (AU)	8.95 (3.12)	5.14 (1.40)	0.12
Total plasma cholesterol (mg/dL)	162 (11.9)	168 (13.2)	0.36
High density lipoprotein cholesterol (mg/dL)	56.6 (5.73)	57.2 (3.49)	0.46
Plasma triglycerides (mg/dL)	80.6 (6.75)	80.6 (9.44)	0.50

*****High blood glucose values are attributed to the terminal nature of the blood collection procedure.

Supplementary Table S2.

The ascending colonic mucosal-associated microbiome was not different between young and old monkeys. This table outlines the lack of significance associated with the first 3 MDS levels at the phylum, class, order, family and genus levels.

		Variance	Age Group	Sequence Depth	Adjusted for Depth │Group
**Taxonomic Level**		**%**	**p-value**	**p-value**	**p-value**
**Phylum**	MDS1	31.03	0.213	0.803	0.621
**Phylum**	MDS2	23.00	0.643	0.002	0.883
**Phylum**	MDS3	13.58	0.235	0.127	0.383
**Class**	MDS1	29.15	0.314	0.021	0.856
**Class**	MDS2	18.89	0.676	0.053	0.837
**Class**	MDS3	11.37	0.563	0.263	0.837
**Order**	MDS1	29.74	0.474	0.001	0.959
**Order**	MDS2	16.41	0.330	0.388	0.630
**Order**	MDS3	11.85	0.657	0.534	0.895
**Family**	MDS1	25.40	0.338	0.000	0.897
**Family**	MDS2	15.07	0.614	0.571	0.852
**Family**	MDS3	11.33	0.328	0.750	0.852
**Genus**	MDS1	24.88	0.557	0.000	0.980
**Genus**	MDS2	14.61	0.972	0.149	0.980
**Genus**	MDS3	11.69	0.502	0.668	0.980

Supplementary Table S3.

The ascending colonic lumen microbiome was not different between young and old monkeys. This table outlines the lack of significance associated with the first 3 MDS levels at the phylum, class, order, family and genus levels.

		Variance	Age Group	Sequence Depth	Adjusted for Depth | Group
**Taxonomic Level**		**%**	**p-value**	**p-value**	**p-value**
**Phylum**	MDS1	41.32	0.194	0.365	0.500
**Phylum**	MDS2	23.58	0.590	0.059	0.780
**Phylum**	MDS3	12.79	0.238	0.784	0.500
**Class**	MDS1	37.71	0.122	0.374	0.469
**Class**	MDS2	19.78	0.702	0.054	0.989
**Class**	MDS3	12.77	0.337	0.251	0.715
**Order**	MDS1	31.72	0.081	0.261	0.566
**Order**	MDS2	20.30	0.516	0.217	0.994
**Order**	MDS3	15.09	0.722	0.024	0.994
**Family**	MDS1	26.90	0.066	0.562	0.437
**Family**	MDS2	17.69	0.943	0.021	0.974
**Family**	MDS3	13.28	0.933	0.149	0.974
**Genus**	MDS1	30.07	0.162	0.917	0.601
**Genus**	MDS2	14.63	0.953	0.002	0.719
**Genus**	MDS3	13.19	0.428	0.902	0.719

Supplementary Table S4.

The fecal microbiome was not different between young and old monkeys. This table outlines the lack of significance associated with the first 3 MDS levels at the phylum, class, order, family and genus levels.

**Taxonomic Level**		**Variance %**	**Age Group p-value**	**Sequence Depth p-value**	**Adjusted for Depth │Group p-value**
**Phylum**	MDS1	57.99	0.146	0.018	0.504
**Phylum**	MDS2	14.22	0.330	0.297	0.504
**Phylum**	MDS3	8.29	0.181	0.533	0.504
**Class**	MDS1	46.12	0.193	0.007	0.557
**Class**	MDS2	15.58	0.262	0.734	0.557
**Class**	MDS3	8.48	0.038	0.313	0.557
**Order**	MDS1	38.52	0.242	0.011	0.682
**Order**	MDS2	15.95	0.186	0.592	0.682
**Order**	MDS3	9.31	0.192	0.104	0.682
**Family**	MDS1	34.78	0.144	0.017	0.493
**Family**	MDS2	11.75	0.330	0.381	0.493
**Family**	MDS3	10.21	0.119	0.240	0.493
**Genus**	MDS1	32.22	0.088	0.019	0.373
**Genus**	MDS2	11.75	0.905	0.532	0.991
**Genus**	MDS3	8.88	0.067	0.804	0.260

Supplementary Table S5.

Breakdown of dietary conditions used in Studies 1, 2, and 3.

Dietary Component	Chow Diet	Western Diet
Total Protein	18.2% of calories	19 % of calories
Total Fat	13.1% of calories	35.5 % of calories
Total Carbohydrates	68.7% of calories	46% of calories
Simple Sugars	5.25% of calories	22% of calories
Cholesterol	0.75 ppm	600ppm
Saturated Fat	0.2% of calories	14% of calories
Sodium	0.72 mg/kCal	0.30 mg/kCal
Crude Fiber	> 6 %	6.1%
Caloric Density	3.43 kcal/g	3.56 kcal/g

Supplementary Table S6.

Basic demographics of young and old monkeys evaluated in Study 2.

	N	Age<br>years	BW<br>kg	Waist<br>cm	Glucose<br>mg/dL	Insulin<br>(µIU/L)	TPC<br>(mg/dL)	ALT<br>(U/L)	ALP<br>(U/L)	LBP-1<br>ng/mL	sCD14<br>ng/mL
Young	6	8.12<br>(1.27)	5.2<br>(0.38)	30.9<br>(2.02)	78.8<br>(6.94)	33.7<br>(15.2)	135<br>(9.46)	58.2<br>(0.93)	83.5<br>(12.4)	2.93<br>(1.15)	2.13<br>(0.09)
Old	12	21.7<br>(0.68)	5.34<br>(0.34)	35.9<br>(1.62)	63.1<br>(6.88)	8.21<br>(2.76)	158<br>(10.0)	49.2<br>(5.83)	92.2<br>(9.79)	3.97<br>(0.78)	2.61<br>(0.13)
p-value		<0.001	0.80	0.04	0.17	0.04	0.08	0.41	0.60	0.53	0.02

Supplementary Table S7.

Old monkeys in Study 2 had elevated baseline markers of microbial translocation (lipopolysaccharide binding protein 1 and soluble CD14 [LBP-1, sCD14]) which did not further elevate following 8 weeks consumption of a Western diet. *p<0.05; #p<0.10 Old vs Young (n=6/group) at baseline. Overall p-value is for ANCOVA.

	**LBP-1 (ng/mL)**		**sCD14 (ng/mL)**	
	**Baseline**	**Post-Diet**	**Baseline**	**Post-Diet**
**Young Western Diet**	2.93 (1.15)	3.73 (1.49)	2.13 (0.09)	1.81 (0.14)
**Old Western Diet**	5.15 (1.17)#	4.88 (1.03)	2.85 (0.16)*	2.46 (0.25)
**p-value**		0.95		0.25

Supplementary Figure S1.

There were significant site differences in the diversity of the microbiome with the ascending colon mucosal microbiome being significantly less diverse than that of feces, or samples from the lumen of the ascending colon ($p=7.35x10^{-6}$).

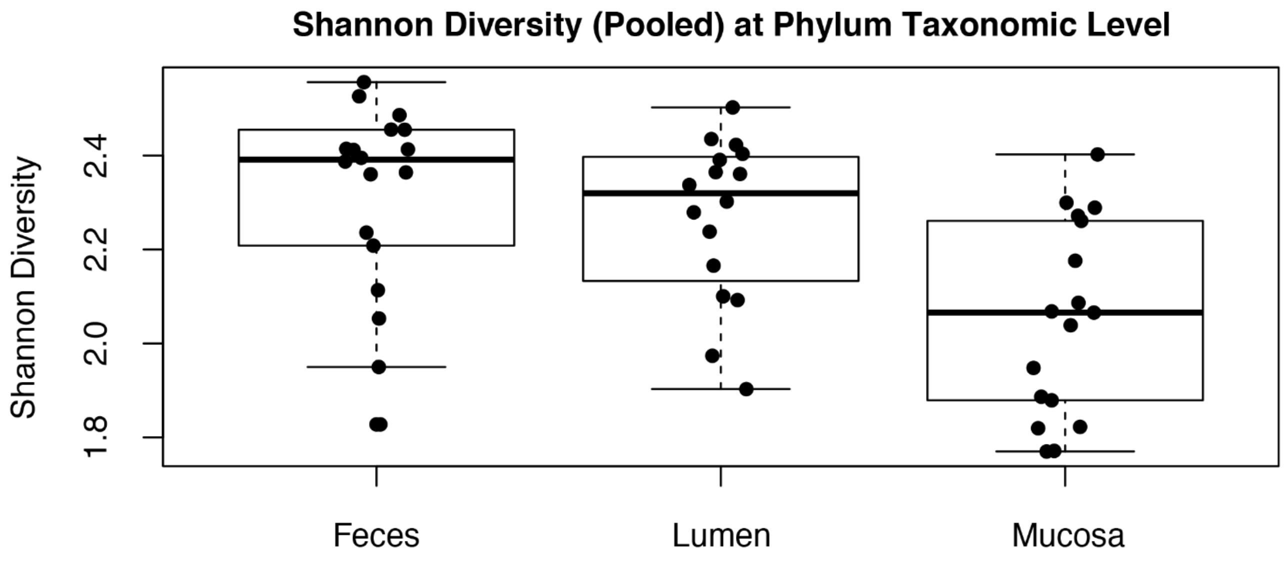

Supplementary Figure S2.

Liver alanine aminotransferase (ALT) levels in monkeys assessed in Study 2 had variable return to baseline values after the Western diet challenge was discontinued for 8 weeks and chow was consumed. Only 40% (2/5) females evaluated returned to baseline and 40% continued to show increasing hepatic damage. The broken line, (----), indicates a median value for age-matched monkeys from the Vervet Research Colony.

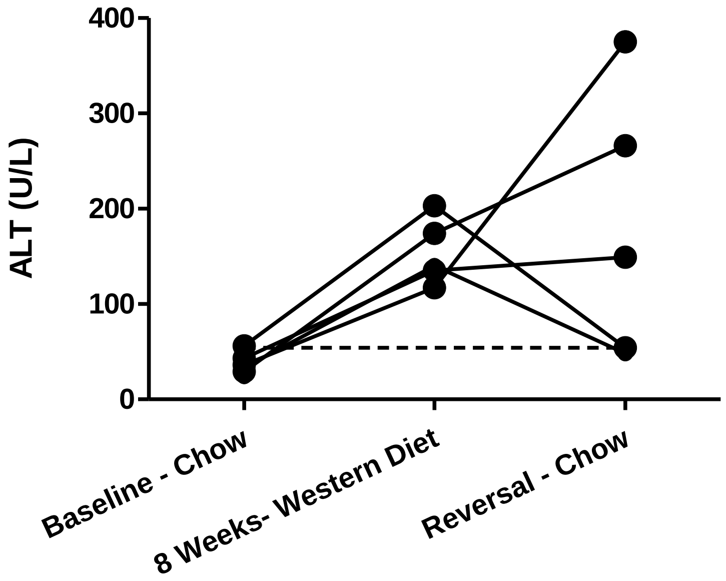

Supplementary Figure S3.

Example histology and measurements from Study 1. Overall structure of the colon is shown in panel A. An example of crypt depth and visualization used to manually count goblet cells shown in panel B. Panel C shows measurement of the muscularis layer thickness.

A.

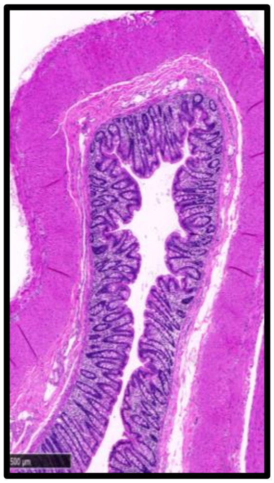

B.

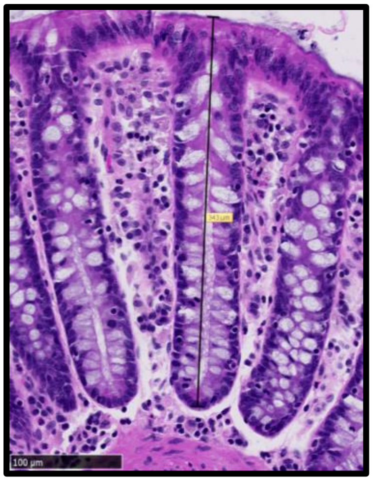

C.

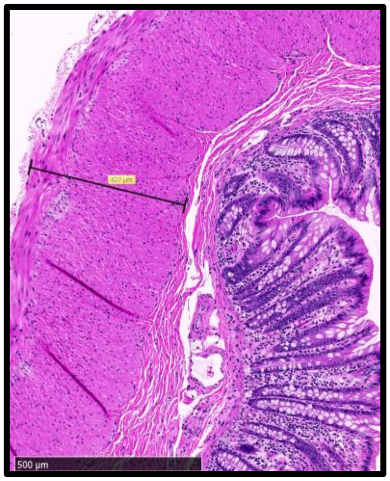

Representative immunostaining for occludin protein abundance is shown in panel D, and claudin-1 in panel E. Co-staining by periodic acid Schiff and Alcian blue for proteoglycan content of mucins is show in panel F.

D.

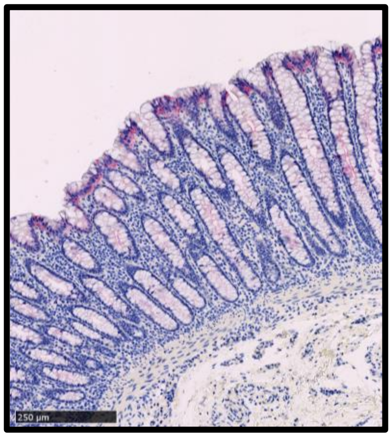

E.

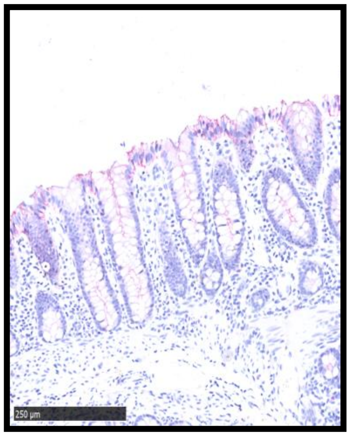

F.

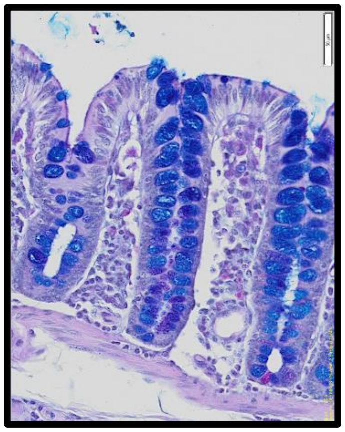

Supplement: Supplementary file 1 — Supplementary Data [file 41598_2018_29473_MOESM1_ESM.pdf]
